# Prognostic Impact of Active Mechanical Circulatory Support in Cardiogenic Shock Complicating Acute Myocardial Infarction, Results from the Culprit-Shock Trial

**DOI:** 10.3390/jcm9061976

**Published:** 2020-06-24

**Authors:** Hans-Josef Feistritzer, Steffen Desch, Anne Freund, Janine Poess, Uwe Zeymer, Taoufik Ouarrak, Steffen Schneider, Suzanne de Waha-Thiele, Georg Fuernau, Ingo Eitel, Marko Noc, Janina Stepinska, Kurt Huber, Holger Thiele

**Affiliations:** 1 Department of Internal Medicine/Cardiology, Heart Center Leipzig at University of Leipzig and Leipzig Heart Institute, 04289 Leipzig, Germany; steffen.desch@medizin.uni-leipzig.de (S.D.); anne.freund@medizin.uni-leipzig.de (A.F.); janine.poess@medizin.uni-leipzig.de (J.P.); Holger.Thiele@medizin.uni-leipzig.de (H.T.); 2 Institut für Herzinfarktforschung GmbH, IHF, 67063 Ludwigshafen, Germany; uwe.zeymer@t-online.de (U.Z.); ouarrak@stiftung-ihf.de (T.O.); schneider@stiftung-ihf.de (S.S.); 3 Klinik für Innere Medizin/Kardiologie, Klinikum 67063 Ludwigshafen, Germany; 4 Department of Cardiology, Angiology and Intensive Care Medicine, University Heart Center Lübeck, University Hospital Schleswig-Holstein, 23538 Lübeck, Germany; Suzanne.deWaha-Thiele@uksh.de (S.d.W.-T.); georg.fuernau@uksh.de (G.F.); Ingo.Eitel@uksh.de (I.E.); 5 University Medical Center Ljubljana, 1000 Ljubljana, Slovenia; marko.noc@mf.uni-lj.si; 6 Institute of Cardiology, 04-628 Warsaw, Poland; janina@stepinska.pl.pl; 7 Department of Cardiology, Wilhelminenspital and Sigmund Freud University, Medical School, 1160 Vienna, Austria; kurt.huber@meduniwien.ac.at

**Keywords:** cardiogenic shock, acute myocardial infarction, mechanical circulatory support, prognosis

## Abstract

Objectives: To analyze the use and prognostic impact of active mechanical circulatory support (MCS) devices in a large prospective contemporary cohort of patients with cardiogenic shock (CS) complicating acute myocardial infarction (AMI). Background: Although increasingly used in clinical practice, data on the efficacy and safety of active MCS devices in patients with CS complicating AMI are limited. Methods: This is a predefined subanalysis of the CULPRIT-SHOCK randomized trial and prospective registry. Patients with CS, AMI and multivessel coronary artery disease were categorized in two groups: (1) use of at least one active MCS device vs. (2) no active MCS or use of intra-aortic balloon pump (IABP) only. The primary endpoint was a composite of all-cause death or renal replacement therapy at 30 days. Results: Two hundred of 1055 (19%) patients received at least one active MCS device (*n* = 112 Impella^®^; *n* = 95 extracorporeal membrane oxygenation (ECMO); *n* = 6 other devices). The primary endpoint occurred significantly more often in patients treated with active MCS devices compared with those without active MCS devices (142 of 197, 72% vs. 374 of 827, 45%; *p* < 0.001). All-cause mortality and bleeding rates were significantly higher in the active MCS group (all *p* < 0.001). After multivariable adjustment, the use of active MCS was significantly associated with the primary endpoint (odds ratio (OR) 4.0, 95% confidence interval (CI) 2.7–5.9; *p* < 0.001). Conclusions: In the CULPRIT-SHOCK trial, active MCS devices were used in approximately one fifth of patients. Patients treated with active MCS devices showed worse outcome at 30 days and 1 year.

## 1. Introduction

Early revascularization significantly improves outcome in patients with cardiogenic shock (CS) complicating acute myocardial infarction (AMI) [1,2]. However, 30-day mortality is still high, ranging between 40 and 50% [3,4,5,6]. The intra-aortic balloon pump (IABP) is one of the oldest mechanical circulatory support (MCS) devices and was frequently used in CS for many decades. The neutral results of the Intra-aortic Balloon Pump in Cardiogenic Shock II (IABP-SHOCK II) trial resulted in a marked decrease in IABP use [7,8,9,10]. In turn, active MCS devices including Impella^®^ (Abiomed, Aachen, Germany), TandemHeart^®^ (CardiacAssist, Inc., Pittsburgh, USA), and veno-arterial extracorporeal membrane oxygenation (ECMO) have been increasingly used in clinical practice during recent years [11,12,13]. Despite the rapid increase in active MCS devices in CS, data derived from randomized controlled trials (RCTs) are scarce. The small IMPRESS-in-Severe-SHOCK trial (*n* = 48) did not show any mortality benefit of Impella CP^®^ compared with IABP [14]. However, this study was markedly underpowered for the 30-day primary endpoint all-cause mortality. A recent matched-pair analysis (237 matched pairs) comparing Impella^®^- and IABP-treated CS patients showed similar 30-day mortality in both groups [15]. In a meta-analysis of RCTs, active MCS devices (*n* = 77) did not result in a mortality benefit but an increased rate of bleeding complications compared with IABP (*n* = 71) [16]. However, outcome data from these RCTs should be interpreted with caution due to the limited statistical power. Regarding veno-arterial ECMO, a meta-analysis of cohort studies suggested a mortality benefit with ECMO compared with IABP in CS patients without cardiac arrest [17]. 

The Culprit Lesion Only PCI versus Multivessel PCI in Cardiogenic Shock (CULPRIT-SHOCK) trial compared two different revascularization strategies in patients with infarct-related CS and multivessel coronary artery disease [18]. Thirty-day and one-year outcome results were previously published [19,20]. In the present subanalysis, we aimed to investigate the use and impact of active MCS devices on clinical outcome.

## 2. Methods

### 2.1. Study Design

The present study is a predefined subanalysis of the randomized CULPRIT-SHOCK trial and prospective CULPRIT-SHOCK registry. In brief, CULPRIT-SHOCK is an investigator-initiated, open-label, European multicenter trial, that randomized patients with infarct-related CS and multivessel coronary artery disease to either culprit lesion-only revascularization with the option of staged revascularization of non-culprit lesions or immediate multivessel revascularization. CS was defined as the presence of hypotension (systolic blood pressure < 90 mmHg for >30 min or catecholamines required to maintain systolic blood pressure >90 mmHg), signs of pulmonary congestion and signs of impaired organ perfusion. Exclusion criteria included resuscitation >30 min, no intrinsic heart action, severe cerebral deficit, need for primary urgent coronary artery bypass grafting, single-vessel disease, mechanical cause of CS, onset of CS > 12 h, severe pulmonary embolism, age >90 years, pregnancy, shock of other cause, severe concomitant disease with life expectancy < 6 months and known severe renal insufficiency (creatinine clearance < 30 mL/kg). The culprit lesion-only strategy resulted in a significant reduction in the primary endpoint of all-cause death or renal replacement therapy at 30 days, with consistent reduction in the composite endpoint at 1-year follow-u-p [19,20]. 

Patients not fulfilling the inclusion criteria or those with exclusion criteria were entered in the prospective CULPRIT-SHOCK registry. For patients unable to consent at the time of randomization, a specific, predefined informed consent process was used [18]. The trial follows the rules of the Declaration of Helsinki. The study protocol was approved by the steering committee and all relevant ethics committees (initial ethic committee: Ethic Committee at the Medical Faculty, Leipzig University; code: 399-12-05112012) [18]. The study was registered at ClinicalTrials.gov (ClinicalTrials.gov Identifier: NCT01927549). 

**Figure 1:**
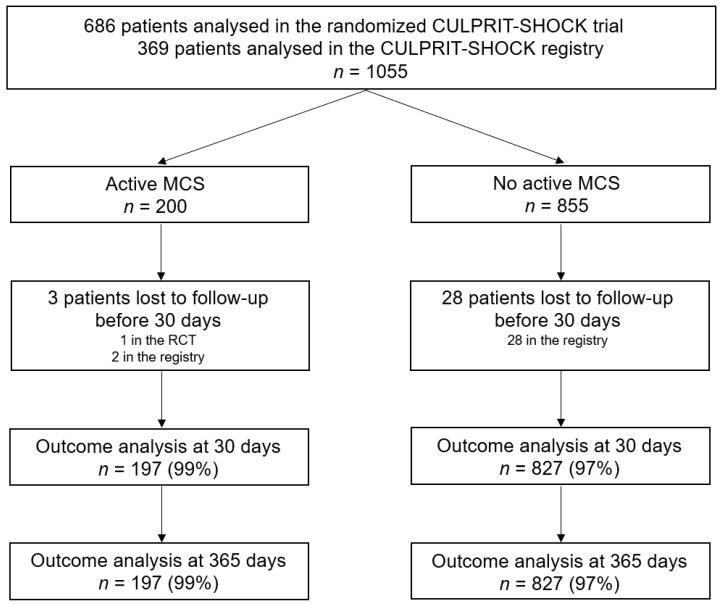
Study flow chart MCS = mechanical circulatory support; RCT = randomized controlled trial.

The study flow chart is presented in Figure 1. The detailed study flow chart including the exclusion rates of initially screened patients was published previously [20]. Patients were categorized according to the use of active MCS devices. Patients treated with at least one active MCS device (e.g., Impella^®^, TandemHeart^®^, ECMO) were assigned to group 1. Patients with no active MCS device or IABP only were assigned to group 2. 

### 2.2. Clinical Endpoints

The primary endpoint of the present analysis was the combination of all-cause death or renal replacement therapy at 30 days. Secondary endpoints included the individual components of the combined endpoint, recurrent myocardial infarction, rehospitalization for congestive heart failure, stroke, repeat revascularization and bleeding at 30 days as well as all-cause mortality at 1 year. Definitions of clinical endpoints were described in detail previously [18]. Bleeding events were categorized according to the Bleeding Academic Research Consortium scale [21]. 

### 2.3. Statistical Analysis

Variables are expressed as a number and corresponding percentages (for categorical variables) or medians and interquartile range (IQR) (for continuous variables). Comparisons between patients with and without active MCS were performed using the chi-square (for categorical variables) or Wilcoxon rank-sum (for continuous variables) test. For outcome analyses, Kaplan–Meier curves with log-rank comparisons were created. Product limit estimator (Kaplan–Meier Estimate) was used to estimate the 1-year mortality. The prognostic impact of MCS was further tested in univariable and multivariable regression analyses. Only variables associated with the use of active MCS in unadjusted analyses (*p* < 0.1) were included in the multivariable model. Accordingly, multivariable adjustment was performed for age, body mass index, diabetes, coronary 3-vessel disease, atrial fibrillation, presence of left bundle branch block, heart rate prior to PCI, plasma creatinine levels and presence of culprit lesion in the right and left main coronary arteries. Plasma glucose levels on admission, arterial lactate prior to PCI and the left ventricular (LV) ejection fraction were not included in the multivariable model because of a high rate of missing values. All tests were 2-tailed and *p*-values < 0.05 were considered statistically significant. Statistical analysis was performed using the SAS software package, version 9.4 (Cary, NC) by independent statisticians at the Institut für Herzinfarktforschung, Ludwigshafen, Germany. 

## 3. Results

### 3.1. Clinical Characteristics

In total, data regarding the use of MCS devices were available in 1055 patients (686 in the randomized trial; 369 in the prospective registry) (Figure 1). At least one active MCS device was used in 200 of 1055 (19%) patients (Table 1). One-hundred-and-twelve (11%) patients were treated with Impella^®^ devices, whereas ECMO was implanted in 95 (9%) patients. In patients treated with ECMO, concomitant IABP was used in 14 of 95 (15%) patients, whereas concomitant Impella^®^ support was used in 8 of 95 (8%) patients. In the no active MCS group, 112 (13%) patients were treated with IABP only.

Baseline clinical characteristics for the total cohort as well as for patients with and without active MCS are summarized in Table 1. Patients treated with active MCS devices were significantly younger, more likely to be diabetics and showed a higher rate of culprit lesions located in the left main coronary artery compared with patients without active MCS (all *p* < 0.05). Heart rate and serum lactate levels measured prior to PCI were significantly higher in the active MCS group, whereas the LV ejection fraction was lower (all *p* < 0.001). The shock index on admission was higher in the active MCS compared with the no active MCS group (0.93, IQR 0.71–1.15 vs. 0.83, IQR 0.65–1.10; *p* = 0.048). High-sensitivity cardiac troponin T levels on admission were similar in patients with and without active MCS (751, IQR 161-5929 ng/L vs. 573, IQR 102–2578 ng/L; *p* = 0.39). Immediate PCI of non-culprit lesions were more frequently performed in patients with active MCS compared with those without active MCS (53% vs. 43%, *p* = 0.01).

### 3.2. Outcome Analysis

Thirty-one (3%) patients were lost to follow-up before 30 days (1 in the randomized trial and 30 in the registry study). Thus, the primary endpoint could be analyzed in 197 patients with active MCS and 827 patients without active MCS (Figure 1). A second patient in the randomized trial and 10 patients in the registry study were additionally lost to follow-up before 1 year. For the survival analysis, these patients were censored at the last date of follow-up. 

**Figure 2:**
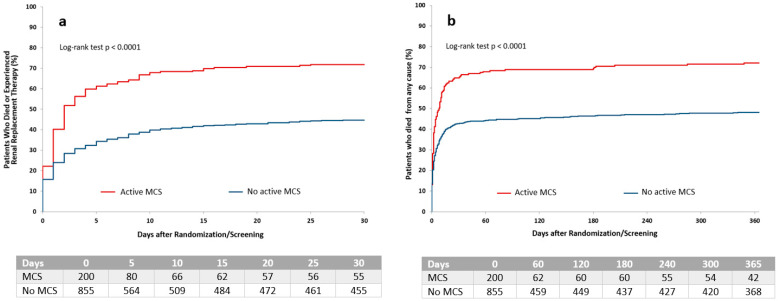
Kaplan–Meier curves of the time to the occurrence of the primary endpoint of all-cause death or renal replacement therapy at 30 days (**a**), and 1-year mortality (**b**). MCS = mechanical circulatory support.

The primary endpoint occurred significantly more often in patients receiving active MCS compared with those without active MCS (142 of 197, 72% vs. 374 of 827, 45%; *p* < 0.001). Secondary endpoints are summarized in Table 2. Kaplan–Meier curves for the primary endpoint and one-year mortality are shown in Figure 2. After the multivariable adjustment, the use of active MCS was still significantly associated with the primary endpoint (odds ratio (OR) 4.0, 95% confidence interval (CI) 2.7–5.9; *p* < 0.001), as well as one-year mortality (OR 3.6, 95% CI 2.4–5.3; *p* < 0.001). Furthermore, the use of active MCS was independently associated with bleeding events at 30 days (OR 3.1, 95% CI 2.1–4.5; *p* < 0.001) (Table 3). 

In patients treated with Impella^®^, ECMO and IABP only, the primary endpoint occurred in 69%, 76% and 42%, respectively. One-year mortality rates were 71%, 71% and 43%.

## 4. Discussion

In the present subanalysis from the randomized CULPRIT-SHOCK trial and CULPRIT-SHOCK registry, we aimed to investigate the prognostic impact of active MCS devices in patients with CS complicating AMI. The major findings were as follows: (1) at least one active MCS device was used in approximately one fifth of patients; (2) the combined endpoint of death and renal replacement therapy occurred more frequently in patients receiving active MCS; and (3) bleeding complications were higher in the active MCS group.

The rationale behind the use of MCS devices in infarct-related CS is the augmentation of cardiac output and arterial blood pressure to improve end-organ perfusion. The neutral results of the IABP-SHOCK II trial resulted in a prompt switch from IABP to more potent active MCS devices in clinical practice [7,8]. These devices, among others, include the microaxial flow pumps from the Impella^®^ family, the TandemHeart^®^ and ECMO [6]. Although frequently used in clinical routine, data supporting a prognostic benefit with active MCS devices in infarct-related CS are limited [14,15,16,22,23,24,25,26,27,28,29,30]. The aim of the present analysis was to investigate the prognostic role of active MCS devices rather than an outcome analysis of patients treated with IABP. Therefore, patients receiving IABP only were assigned to the control group. 

## 5. Clinical Characteristics and Rates of MCS Implementation

With the exception of the IMPRESS-in-Severe-SHOCK trial, which included younger patients, the age of included patients in previous RCTs of active MCS was similar to the present study [14,22,23,24]. On the other hand, previous cohort studies showed a lower age of patients receiving active MCS, which might be due to selection bias [17,27,31,32]. The shock index, which was identified as a strong outcome predictor in STEMI patients, was higher in patients receiving active MCS [33]. Arterial lactate levels measured prior to PCI were significantly higher in patients treated with active MCS devices. These findings might reflect the more severely impaired hemodynamics and subsequent end-organ perfusion in these patients. In line, patients in the active MCS group showed a lower LV ejection fraction. 

In the present study, at least one active MCS device was inserted in 19% of patients, whereas IABP was used in 13% in the no active MCS group. Published data regarding the frequency of MCS device implantation in infarct-related CS are conflicting. In a large US registry, IABP and active MCS devices were used in 39% and 3.5% of CS patients between 2009 and 2013 [34]. Whereas the proportion of IABP declined over time, the use of active MCS remained unchanged. In patients undergoing PCI with MCS, Impella^®^ was used in 9.9% of patients between 2004 and 2016 in the US [29]. The frequency of Impella^®^ use increased over time, reaching 31.9% in 2016. Another US registry reported rates of 72%, 17% and 11% for IABP, Impella^®^ and ECMO support between 2017 and 2018 [27]. According to a recent report from a large registry study from Switzerland, Impella^®^ or ECMO are used in approximately 10% of patients with infarct-related CS, with increasing rates during the last seven years [35]. On the other hand, rates of IABP insertion (27%) remained unchanged during the last decade. A Danish registry reported an increasing use of Impella^®^ (3% to 16%) and ECMO (0% to 6%) between 2010 and 2017 [36]. Conversely, the use of IABP significantly decreased during the same period (35% to 0.4%).

The use of ECMO is associated with an increase in LV afterload which may further aggravate myocardial ischemia, LV dysfunction, pulmonary congestion and the risk of LV thrombus formation [37,38,39]. These effects can be counteracted by the use of LV unloading strategies. According to a recent meta-analysis, concomitant LV unloading is implemented in 42% of adult CS patients treated with ECMO [40]. Notably, with rates > 90%, IABP was by far the most frequently used device to achieve LV unloading. In the present study, concomitant IABP or Impella^®^ were used in 15% and 8% of patients treated with ECMO. However, due to the lack of RCTs in this field, the use of LV unloading strategies in ECMO-treated patients is mainly based on local clinical practice and the discretion of the treating physician. 

## 6. Clinical Outcome

Data on the prognostic impact of active MCS devices in infarct-related CS are limited. There are several small RCTs comparing active MCS devices (TandemHeart^®^ in two trials; Impella^®^ in two trials) with IABP [14,22,23,24]. According to a meta-analysis of these trials, the use of active MCS showed similar 30-day mortality compared to IABP (45.5% vs. 45.1%) [16]. In line, a large matched-pair analysis as well as a retrospective cohort study showed similar 30-day mortality in Impella^®^- and IABP-treated patients [15,25]. 

More recent registry studies from the US even showed a higher mortality with Impella^®^ use in comparison with a control which persisted after adjustment and in the propensity matching [26,29,41]. This is in line with our study, where the primary endpoint as well as 30-day and one-year mortality more frequently occurred in patients treated with active MCS devices. Our findings might be due to some selection bias, as active MCS devices might be more frequently implanted in patients presenting a worse hemodynamic condition. However, worse outcome with active MCS was still detected after adjustment for the patients’ risk profiles, comorbidities and angiographic findings. Albeit, the comparison of our results with the above-mentioned outcome data is hampered since only 13% of patients in our control group were treated with IABP.

Large-scale randomized trials investigating the prognostic role of ECMO support in CS are lacking so far. A meta-analysis of cohort studies comparing ECMO and IABP showed significantly lower 30-day mortality with ECMO (38% vs. 70%) [17]. A recent small randomized trial in CS comparing ECMO with a control showed a similar 30-day mortality and a similar LV ejection fraction as the primary endpoint [30]. In our study, numerical event rates of the primary endpoint and one-year mortality were similar in ECMO- and Impella^®^-treated patients. On the other hand, better outcome was observed for patients treated with IABP only. Since the more potent active MCS devices might be preferably implanted in patients with worse hemodynamics, this finding to some extent might be due to selection bias. 

In line with previous data, the use of active MCS devices was significantly associated with bleeding complications in the present study [15,16]. The major prognostic role of bleeding complications in infarct-related CS was recently confirmed in another subanalysis of the CULPRIT-SHOCK trial [42]. According to our and previous data, further studies are needed to identify high-risk CS patients who benefit from active MCS devices.

## 7. Study Limitations

Some study limitations should be mentioned. Although worse outcome with active MCS was observed even after the multivariable adjustment, our findings might be influenced by selection bias. Furthermore, a multivariable adjustment for lactate levels could not be performed due to a high number of missing values. Second, the time point of MCS device implantation was not systematically assessed in the present study. Third, due to the limited number of patients receiving a second MCS device in addition to ECMO, the prognostic role of LV unloading strategies could not be assessed in the present study. 

## 8. Conclusions

In the present subanalysis of the CULPRIT-SHOCK randomized trial and prospective registry, at least one active MCS device was implanted in 19% of patients with CS complicating AMI. Compared with patients without active MCS devices, those treated with active MCS showed worse outcome at 30-day and one-year follow-up. Therefore, randomized trials are needed to define the value of MCS in CS. 

## Figures and Tables

**Table 1:** Baseline characteristics.

	Total Study (*n* = 1055)	Active MCS (*n* = 200)	No Active MCS (*n* = 855)	*p*-Value
Age, years (*n* = 1055)	68 (59–77)	66 (57–75)	69 (59–77)	0.004
Male gender, *n* (%)	778/1038 (75)	158/200 (79)	620/838 (74)	0.14
Body mass index, kg/m² (*n* = 970)	27 (25–29)	27 (25–30)	26 (24–29)	0.04
Cardiovascular risk factors, *n* (%)				
Current smoking	296/985 (30)	58/189 (31)	238/796 (30)	0.83
Hypertension	602/1001 (60)	115/194 (59)	487/807 (60)	0.78
Dyslipidemia	335/999 (34)	61/193 (32)	274/806 (34)	0.53
Diabetes mellitus	302/1001 (30)	71/193 (37)	231/808 (29)	0.03
Medical history, *n* (%)				
Prior myocardial infarction	167/1005 (17)	30/193 (16)	137/812 (17)	0.66
Prior PCI	180/1005 (18)	38/193 (20)	142/812 (17)	0.47
Prior CABG	50/1010 (5)	10/194 (5)	40/816 (5)	0.88
Prior stroke	76/1008 (8)	13/194 (7)	63/814 (8)	0.62
Peripheral artery disease	112/1009 (11)	16/194 (8)	96/815 (12)	0.16
Chronic renal insufficiency	63/1006 (6)	16/194 (8)	47/812 (6)	0.20
Hemodynamic parameters on admission, *n* (%)				
Heart rate, bpm (*n* = 990)	91 (72–109)	99 (80–112)	90 (70–108)	<0.001
Systolic blood pressure, mmHg (*n* = 881)	100 (84–124)	102 (80–125)	100 (85–123)	0.99
Diastolic blood pressure, mmHg (*n* = 867)	60 (50–79)	63 (50–80)	60 (50–78)	0.38
Resuscitation before randomization, *n* (%)	550/1015 (54)	108/196 (55)	442/819 (54)	0.77
Arterial lactate pre-PCI, mmol/l (*n* = 609)	5.2 (2.7–8.6)	6.5 (3.5–11.0)	4.9 (2.5–7.9)	<0.001
pH < 7.36 on admission, *n* (%)	609/988 (62)	123/192 (64)	486/796 (61)	0.44
Coronary 3-vessel disease, *n* (%)	573/1031 (56)	123/200 (62)	450/831 (54)	0.02
Culprit lesion, *n* (%)				
RCA	292/1028 (28)	44/200 (22)	248/828 (30)	0.03
LM	82/1028 (8)	26/200 (13)	56/828 (7)	0.003
LAD	443/1028 (43)	87/200 (44)	356/828 (43)	0.90
LCX	199/1028 (19)	42/200 (21)	157/828 (19)	0.51
Bypass	12/1028 (1.1)	1/200 (0.5)	11/828 (1.3)	0.33
Angiographic parameters, *n* (%)				
TIMI flow 0 pre-PCI culprit lesion, *n* (%)	577/1016 (57)	116/199 (58)	461/817 (56)	0.55
TIMI flow 3 post-PCI culprit lesion, *n* (%)	854/1022 (84)	168/199 (84)	686/823 (83)	0.64
PCI and stent implantation culprit lesion, *n* (%)	952/1033 (92)	192/200 (96)	760/833 (91)	0.02
Immediate PCI of non-culprit lesions, *n* (%)	466/1036 (45)	106/200 (53)	360/836 (43)	0.01
Functional parameters				
LV ejection fraction, % (*n* = 374)	32 (25–40)	28 (20–40)	35 (25–44)	<0.001
Mechanical support system used, *n* (%)				
Any, *n* (%)	312/1055 (30)	200/200 (100)	112/855 (13)	<0.001
IABP	129/1055 (12)	17/200 (9)	112/855 (13)	<0.001
Impella 2.5	44/1055 (4)	44/200 (22)	0/855 (0)	-
Impella 5.0	0/1055 (0)	0/200 (0)	0/855 (0)	-
Impella CP	68/1055 (6)	68/200 (34)	0/855 (0)	-
TandemHeart	2/1055 (0.2)	2/200 (1)	0/855 (0)	-
ECMO	95/1055 (9)	95/200 (48)	0/855 (0)	-
Other	4/1055 (0.4)	4/200 (2)	0/855 (0)	-
Time to hemodynamic stabilization, days (*n* =897)	3 (1–6)	3 (1–9)	3 (1–5)	0.02
Mild induced hypothermia, *n* (%)	326/1031 (32)	63/200 (32)	263/831 (32)	0.97

CABG = coronary artery bypass graft; ECMO = extracorporeal membrane oxygenation; IABP = intra-aortic balloon pump; LAD = left anterior descending coronary artery; LCX = left circumflex coronary artery; LM = left main coronary artery; LV = left ventricular; MCS = mechanical circulatory support; PCI = percutaneous coronary intervention; RCA = right coronary artery; TIMI = thrombolysis in myocardial infarction.

**Table 2:** Clinical outcome for patients with and without active mechanical circulatory support.

	Total Study (*n* = 1024)	Active MCS (*n* = 197)	No Active MCS (*n* = 827)	*p*-Value
All-cause death or renal replacement therapy at 30 days, *n* (%)	516/1024 (50)	142/197 (72)	374/827 (45)	<0.001
All-cause death at 30 days, *n* (%)	487/1024 (48)	129/197 (65)	358/827 (43)	<0.001
Renal replacement therapy at 30 days, *n* (%)	126/1024 (12)	54/197 (27)	72/827 (9)	<0.001
Myocardial reinfarction at 30 days, *n* (%)	11/1024 (1)	2/197 (1)	9/827 (1)	0.93
Rehospitalization for congestive heart failure at 30 days, *n* (%)	4/1024 (0.4)	0/197 (0)	4/827 (0.5)	0.33
Stroke at 30 days, *n* (%)	35/1024 (3)	12/197 (6)	23/827 (3)	0.02
Repeat revascularization at 30 days, *n* (%)	101/1024 (10)	23/197 (12)	78/827 (9)	0.34
Bleeding at 30 days, *n* (%)	196/1024 (19)	72/197 (37)	124/827 (15)	<0.001
All-cause death at 1 year, product limit estimator (%)		72	48	<0.001

MCS = mechanical circulatory support. Bold type indicates statistical significance (*p* < 0.05).

**Table 3:** Multivariable regression analyses for the association of MCS with primary and secondary endpoints.

	OR (95% CI) *	*p*-Value
All-cause death or renal replacement therapy at 30 days	4.0 (2.7–5.9)	<0.001
All-cause death at 30 days	3.1 (2.1–4.6)	<0.001
Renal replacement therapy at 30 days	3.8 (2.4–5.9)	<0.001
Stroke at 30 days	3.0 (1.4–6.7)	0.01
Bleeding at 30 days	3.1 (2.1–4.5)	<0.001
All-cause death at 1 year	2.1 (1.6–2.5) **	<0.001

CI = confidence interval; OR = odds ratio. * adjusted for: age, body mass index, diabetes, coronary 3-vessel disease, atrial fibrillation, presence of left bundle branch block, heart rate prior to percutaneous coronary intervention, plasma creatinine levels and presence of culprit lesion in the right and left main coronary arteries. ** Hazard ratio (HR) (95% confidence interval (CI)).

## Abbreviations

AMI	acute myocardial infarction
CS	cardiogenic shock
ECMO	extracorporeal membrane oxygenation
IABP	intra-aortic balloon pump
IQR	interquartile range
LV	left ventricular
MCS	mechanical circulatory support
PCI	percutaneous coronary intervention
RCT	randomized controlled trial
